# Time-frequency analysis of event-related brain recordings: Effect of noise on power

**DOI:** 10.1016/j.heliyon.2024.e35310

**Published:** 2024-09-06

**Authors:** Guillaume Marrelec, Jonas Benhamou, Michel Le Van Quyen

**Affiliations:** Laboratoire d'Imagerie Biomédicale, LIB, Sorbonne Université, CNRS, INSERM, F-75006, Paris, France

**Keywords:** Electroencephalography (EEG), Brain rhythms, Time-frequency transform, Power, Noise, Color noise, High frequency oscillations (HFOs)

## Abstract

In neuroscience, time-frequency analysis is widely used to investigate brain rhythms in brain recordings. In event-related protocols, it is applied to quantify how the brain responds to a stimulation repeated over many trials. We here focus on two common measures: the power of the transform for each single trial averaged across trials, avgPOW; and the power of the transform of the average evoked potential, POWavg. We investigate the influence of additive noise on these two measures. We quantify the expected effect using theoretical calculations, simulated data and experimental brain recordings. We also consider the case of color noise. We extract the main factors influencing the effect of noise on POWavg and avgPOW, such as the noise variance, the number of trials, the sampling rate, the type of noise, the type of time-frequency transform and the frequency of interest. When dealing with time-frequency analysis, the impact of noise on the neuroscientist's work can drastically vary depending on these factors. The present results should help researchers improve their understanding and interpretation of time-frequency diagrams, as well as optimize their experimental designs and analyses based on their neuroscientific question.

## 1 Introduction

In neuroscience, where one is interested in brain rhythms, time-frequency (TF) methods have extensively been used for the analysis of in-vivo brain recording techniques [65], [18], [37], including electroencephalography (EEG), magnetoencephalography (MEG), intracranial EEG (iEEG), and microelectrode recordings (measuring local field potentials, LFPs) [4], [58], [27], [8], [36], [22]. A standard procedure is the so-called event-related protocol, where one records how the brain responds to a given stimulation over many trials. The signal measured in response to one stimulation is called an event-related response. Studies have shown that time-frequency analyses are a powerful means to detect transient bursts of high-frequency (>40 Hz) activity in response to sensory stimulation [23], [53], [14], [15]. The origin of these activations is still a subject of discussion, as they may result from two distinct phenomena: They can either be tightly time-locked to the stimulus (stimulus-evoked neuronal activity) or phase-locked to the stimulus (stimulus-induced oscillations) [60]. Short-latency oscillations have been found in the average evoked potential within 100 ms of stimulus onset [54], [42], [41], [17]. By contrast, stimulus-induced oscillations disappear in the average evoked potential because of the jitter in latency from one trial to the next [63]. As a consequence, they have to be extracted using methods that are able to distinguish between phase-locked and non-phase-locked activity.

To be able to discriminate between stimulus-evoked and stimulus-induced oscillations, two main measures of power have been considered. Starting from a collection of *N* signals $x_{n}(t)$, n=1,…,N, acquired from *N* trials, a first measure is the amplitude $\left|T_{x_{n}}(t,f)\right|$, or the power $|T_{x_{n}}(t,f){|}^{2}$, of the transform for each single trial *n* averaged across trials [30, Chap. 9]

(1) $$\mathrm{avgPOW}=\frac{1}{N}\sum_{n=1}^{N}{\left|T_{x_{n}}(t,f)\right|}^{2}.$$

A second measure is the power of the time-frequency transform applied to the average evoked potential [60]

(2) $$\mathrm{POWavg}={\left|T_{\bar{x_{n}}}(t,f)\right|}^{2}.$$

These two measures are sensitive to different aspects of event-related responses: evoked responses for avgPOW, and phase resetting for POWavg [60], [41]. In [5], we refined the relationship between the two measures in the absence of noise using calculations and simulations.

Following this work, we wondered what the influence of noise on the relationship could be. To answer this question, we first needed to better understand the effect of noise on avgPOW and POWavg. Indeed, signals from brain recordings are composed of a part that is relevant to brain activity, and another part that has other origin, and is therefore considered as noise. Experimentally, it has been observed that POWavg is quite robust to noise compared to avgPOW; also both measures appear to be differentially affected by the temporal properties of the signal. Using theoretical calculations, simulation studies, and analysis of experimental data, we here investigate the effect of additive white Gaussian noise (AWGN) on the expected values of avgPOW and POWavg. Using general calculations, we first show that this effect is additive and positive for both measures, and increases with increasing noise variance. By contrast, we also show that the number of trials has an effect on POWavg but not on avgPOW. We also investigate the important case of a noise that is wide-sense stationary with power spectral density of the form $1/f^{c}$, also known as color noise. We illustrate these general results in the particular case of an oscillatory signal analyzed with the S-transform [59]. We also confirm the predicted behaviors on simulated data as well as experimental brain recordings.

The outline of the manuscript is the following. In Section 2, we provide the general theoretical developments, which are then illustrated on an oscillatory signal in Section 3. We investigate synthetic data in Section 4. Section 5 is devoted to the analysis of experimental data. Further issues are discussed in Section 6.

## 2 Theoretical developments

In this section, we investigate the theoretical implications of considering noisy signals for POWavg and avgPOW. We first introduce time-frequency transform and the S-transform (Section 2.1). We set the model of noisy data with additive noise (Section 2.2). We then investigate the statistical properties of the time-frequency transform of noise (Section 2.3) and then quantify the effect of noise on POWavg and avgPOW (Section 2.4). We finally consider the influence of the type of noise (Section 2.5).

### 2.1 Time-frequency analysis and the S-transform

Time-frequency analysis is a generic approach for the analysis of signals whose frequency content are deemed meaningful but nonstationary [11], [59], [21], [43], [28], [1]. All methods map a one-dimensional real or complex signal s(t) into a two-dimensional complex-valued function $T_{s}(t,f)$ that can be expressed in the following general form

(3) $$T_{s}(t,f)=\int s(u)\;\phi_{t,f}{\left(u\right)}^{⁎}\;du.$$

$\left|T_{s}(t,f)\right|$, $|T_{s}(t,f){|}^{2}$, and $\arg[T_{s}(t,f)]$ are respectively the amplitude (or modulus), power, and phase (or argument) of the time-frequency transform at time *t* and frequency *f*.

The S-transform [59] is a type of time-frequency transform that is commonly used in the analysis of brain recordings. It is a type of time-frequency transform that acts as a band-pass filter or a windowed Fourier transform with a Gaussian window whose width decreases with increasing frequency (standard deviation 1/|f|). Since we deal with real signals, we assume that, for each signal s(u), the S-transform is applied to the analytic signal $s_{a}(u)$. See Section II of Supplementary Material for more details. For f>0, this is tantamount to using the following formula for the transform

$$T_{s}(t,f)=\left|f\right|\sqrt{\frac{2}{\pi }}\int s(u)\;e^{-\frac{1}{2}f^{2}{\left(u-t\right)}^{2}}e^{-2i\pi fu}\;du.$$

We can express this equation as in (3) with

(4) $$\phi_{t,f}(u)=\left|f\right|\sqrt{\frac{2}{\pi }}\;e^{-\frac{1}{2}f^{2}{\left(u-t\right)}^{2}}e^{2i\pi fu}.$$

The time-frequency transform is linear: For any two signals $s_{1}(t)$ and $s_{2}(t)$, and real numbers $\lambda_{1}$ and $\lambda_{2}$, we have

(5) $$T_{\lambda_{1}s_{1}+\lambda_{2}s_{2}}(t,f)=\lambda_{1}T_{s_{1}}(t,f)+\lambda_{2}T_{s_{2}}(t,f).$$

In the case of *N* signals $s_{n}(t)$, n=1,…,N, with average

(6) $$\bar{s_{n}}(t)=\frac{1}{N}\sum_{n=1}^{N}s_{n}(t),$$

this entails

(7) $$T_{\bar{s_{n}}}(t,f)=\frac{1}{N}\sum_{n=1}^{N}T_{s_{n}}(t,f).$$

### 2.2 Model of noisy data

In the following, we consider *N* signals $x_{n}(t)$, n=1,…,N, where each $x_{n}(t)$ can be decomposed into the sum of a signal of interest $s_{n}(t)$ and a noise component $b_{n}(t)$,

(8) $$x_{n}(t)=s_{n}(t)+b_{n}(t).$$

In this expression, the $s_{n}(t)$'s are assumed to be *N* independent and identically distributed (i.i.d.) realizations of a signal of interest s(t), and the $b_{n}(t)$'s are *N* realizations of a noise b(t) with zero mean and variance $\sigma^{2}$.

Akin to (6), we denote by $\bar{x_{n}}(t)$ and $\bar{b_{n}}(t)$ the average measured signal and the average noise, respectively. By averaging (8), we obtain

(9) $$\bar{x_{n}}(t)=\bar{s_{n}}(t)+\bar{b_{n}}(t).$$

### 2.3 Time-frequency transform of noise

To investigate the effect of noise on the time-frequency transform, we need to consider the time-frequency transform of (8). However, this requires taking the time-frequency transform of the noise component, a step that is not mathematically straightforward, since time-frequency transform is defined for a continuous function while Gaussian noise (and, in particular, white Gaussian noise) is often assumed to be a discrete process. A potential solution would be to consider a white noise process [38], and another one would involve a detour into the Schwartz space of rapidly decaying smooth complex valued functions of a real variable [3].

Note however that this is only a theoretical problem. In practice, any routine for time-frequency transform takes discrete signals as inputs and approximates integrals with sums. In particular, we here use an approximation in terms of Riemann sums,

(10) $$T_{b}(t,f)\approx T_{b}^{\mathrm{RS}}(t,f)=\delta t\sum_{k}b(u_{k})\;\phi_{t,f}^{⁎}(u_{k}),$$

where *δt* is the sampling rate, and b(t) is assumed to be sampled at times $u_{k}=u_{0}+k\delta t$. From (8), we can show that the linearity of the time-frequency transform is valid, i.e., (see Appendix A)

(11) $$T_{x_{n}}(t,f)=T_{s_{n}}(t,f)+T_{b_{n}}(t,f)$$

and

(12) $$T_{\bar{x_{n}}}(t,f)=T_{\bar{s_{n}}}(t,f)+T_{\bar{b_{n}}}(t,f).$$

Also, since the $b_{n}$'s are i.i.d. for n=1,…,N, so are their time-frequency transforms.

The statistical properties of $T_{b}(t,f)$ are not straightforward either. For now, we calculate $E\left[T_{b}(t,f)\right]$, the expectation of $T_{b}(t,f)$, which can be obtained through the Riemann approximation (see Appendix A)

(13) $$E\left[T_{b}(t,f)\right]=0.$$

Note that this value does not depend on the type of noise nor on the time-frequency transform used.

### 2.4 Effect of noise on POWavg and avgPOW

We are now in position to investigate the effect of noise on the time-frequency transform of x(t). We first consider POWavg. Using its definition, (2), the linearity of the time-frequency transform, (11), the independence of the $s_{n}$'s and $b_{n}$'s, and the fact that $E[T_{b}(t,f)]=0$, (13), we show that $E\left[{\mathrm{POWavg}}_{x_{1:N}}(t,f)\right]$ can be expressed as (see Appendix B)

(14) $$E\left[{\mathrm{POWavg}}_{x_{1:N}}(t,f)\right]=E\left[{\mathrm{POWavg}}_{s_{1:N}}(t,f)\right]+\frac{1}{N}E\left[{\left|T_{b}(t,f)\right|}^{2}\right].$$

This shows that, in the presence of noise, $E[{\mathrm{POWavg}}_{x_{1:N}}(t,f)]$ differs from $E[{\mathrm{POWavg}}_{s_{1:N}}(t,f)]$ by a quantity that is equal to $E[|T_{b}(t,f){|}^{2}]/N$. The two main consequences of this result are:

• The expected effect of noise is to systematically overestimate POWavg;
• The noise is expected to have vanishing influence with an increasing number of trials.

In a similar fashion, we can investigate the influence of noise on avgPOW. Using its definition, (1), and the same properties as above, we can show that $E\left[{\mathrm{avgPOW}}_{x_{1:N}}(t,f)\right]$ can be expressed as (see Appendix B)

(15) $$E\left[{\mathrm{avgPOW}}_{x_{1:N}}(t,f)\right]=E\left[{\mathrm{avgPOW}}_{s_{1:N}}(t,f)\right]+E\left[{\left|T_{b}(t,f)\right|}^{2}\right].$$

As a consequence, we have the two following properties:

• Akin to POWavg, the expected effect of noise is to systematically overestimate avgPOW;
• Unlike POWavg, the noise has expected constant, non-vanishing influence on avgPOW, regardless of the number of trials.

### 2.5 Case of color noise

According to (14) and (15), noise influences the expected values of avgPOW and POWavg through $E[|T_{b}(t,f){|}^{2}]$, which is the second order statistic of the time-frequency transform of the noise. This quantity is more complex to calculate than the first-order statistic (i.e., the expectation). Its value depends on the type of noise and the time-frequency transform considered. It has been investigated by various authors, both from a theoretical and a numerical perspective in the particular case of Gaussian white (i.e., i.i.d.) noise [62], [24], [2], [67], [25], [3] and in the more general setting of stationary zero-mean Gaussian noise [38]. In particular, there has been a debate whether the sampling rate has an influence in the expression of the variance of $T_{b}(t,f)$ [24], [67], [25].

In the following, we consider wide-sense stationary noise, i.e., noise for which the mean and variance are time independent. Using a derivation similar to [38], it can be shown that $E[|T_{b}(t,f){|}^{2}]$ has a general expression of the form (see Section III-A of Supplementary Material)

(16) $$E\left[{\left|T_{b}(t,f)\right|}^{2}\right]=\int S_{b}(\nu ){\left|\overset{ˆ}{\phi_{t,f}}(\nu )\right|}^{2}d\nu .$$

In this expression, $\overset{ˆ}{\phi_{t,f}}(\nu )$ is the Fourier transform of $\phi_{t,f}$ as defined in (3). $S_{b}(\nu )$ is the power spectral density (PSD) of the noise. It describes the frequency content of the noise along the various frequencies.

In the case of white noise with variance $\sigma^{2}$, the PSD is given by

(17) $$S_{b}(\nu )=\sigma^{2}\;\delta t,$$

so that

(18) $$E\left[{\left|T_{b}(t,f)\right|}^{2}\right]=\sigma^{2}\;\delta t\int {\left|\overset{ˆ}{\phi_{t,f}}(\nu )\right|}^{2}d\nu =\sigma^{2}\;\delta t\int {\left|\phi_{t,f}(u)\right|}^{2}du.$$

The value of the integral depends on the type of time-frequency transform considered. In the case of the S-transform, we obtain (see Section III-B of Supplementary Material)

(19) $$E\left[{\left|T_{b}(t,f)\right|}^{2}\right]=\frac{|f|\sigma^{2}\;\delta t}{\sqrt{\pi }},$$

which is a linear function of *f*. Note that, regarding the above-mentioned debate about the effect of the sampling rate, the present derivation supports the conclusion that it actually does have an influence.

Besides white noise, it is sometimes important to consider models of noise with temporal correlation. A usual such model is color noise, i.e., noise whose spectral power density $S_{b}(\nu )$ is approximately proportional to $1/\nu^{c}$ for *ν* departing from 0.

For a color noise, $E[|T_{b}(t,f){|}^{2}]$ can be approximated as

(20) $$E\left[{\left|T_{b}(t,f)\right|}^{2}\right]\propto \int \frac{1}{\nu^{c}}\;{\left|\overset{ˆ}{\phi_{t,f}}(\nu )\right|}^{2}d\nu .$$

For the S-transform, we obtain (see Section III-C of Supplementary Material)

(21) $$E\left[{\left|T_{b}(t,f)\right|}^{2}\right]\propto \frac{1}{f^{c-1}}.$$

As a consequence, the variance of the noise time-frequency transform decays slower than its power spectral density ($1/f^{c-1}$ instead of $1/f^{c}$). Note that the result obtained for white noise, (19), is compatible with this result with c=0 (which indeed corresponds to white noise). Also, the exact expression depends on the type of process used to generate the noise, i.e., on the exact expression of $S_{b}(\nu )$.

## 3 Oscillatory signal

To provide a detailed illustration of the effect of noise, we consider the case of a real-valued oscillatory signal with Gaussian noise analyzed with the S-transform. The model is introduced in Section 3.1. The expressions for the S-transform, avgPOW and POWavg are given in Sections 3.2, 3.3 and 3.4, respectively. We finally give a numerical example in Section 3.5. A summary of calculated results is given in Table 1.

**Table 1:** **Effect of noise on avgPOW and POWavg for real-valued oscillatory signal.** Summary of results for the S-transform. *O*(⋅) is the standard Bachmann–Landau notation.

Quantity	Expression
$T_{s_{n}}(t,f)$	$\Omega_{n}e^{-\frac{1}{2}{\left(2\pi \right)}^{2}{\left(1-\frac{\nu_{0}}{f}\right)}^{2}}e^{i[\phi_{n}-2\pi (f-\nu_{0})t]}$
${\left|T_{s_{n}}(t,f)\right|}^{2}$	$\Omega_{n}^{2}e^{-{\left(2\pi \right)}^{2}{\left(1-\frac{\nu_{0}}{f}\right)}^{2}}$
${\mathrm{avgPOW}}_{s_{1:N}}(t,f)$	$e^{-{\left(2\pi \right)}^{2}{\left(1-\frac{\nu_{0}}{f}\right)}^{2}}\frac{1}{N}\sum_{n=1}^{N}\Omega_{n}^{2}$
$E\left[{\mathrm{avgPOW}}_{s_{1:N}}(t,f)\right]$	$\left(\Omega_{0}^{2}+\tau_{\Omega }^{2}\right)e^{-{\left(2\pi \right)}^{2}{\left(1-\frac{\nu_{0}}{f}\right)}^{2}}$
${\mathrm{POWavg}}_{s_{1:N}}(t,f)$	$e^{-{\left(2\pi \right)}^{2}{\left(1-\frac{\nu_{0}}{f}\right)}^{2}}{\left|\frac{1}{N}\sum_{n=1}^{N}\Omega_{n}e^{i\phi_{n}}\right|}^{2}$
$E\left[{\mathrm{POWavg}}_{s_{1:N}}(t,f)\right]$	$\Omega_{0}^{2}\rho^{2}e^{-{\left(2\pi \right)}^{2}{\left(1-\frac{\nu_{0}}{f}\right)}^{2}}+O\left(\frac{1}{N}\right)$

### 3.1 Model

We consider a model in which we observe *N* repetitions of a real-valued oscillatory signal with constant frequency $\nu_{0}$ and varying amplitude $\Omega_{n}$ and phase $\phi_{n}$

(22) $$s_{n}(t)=\Omega_{n}\cos(2\pi \nu_{0}t+\phi_{n}),\;n=1,\ldots ,N.$$

We assume that the $\Omega_{n}$'s are i.i.d. repetitions of $\Omega ∼N(\Omega_{0},\tau_{\Omega }^{2})$, while the $\phi_{n}$'s are i.i.d. repetitions of $\phi ∼\mathrm{VonMises}(\phi_{0},\kappa )$. Furthermore, $\Omega_{n}$ and $\phi_{n}$ are assumed to be independent from each other for every *n*. We define the circular mean as [44, §3.4.2]

(23) $$E(e^{i\phi })=\rho e^{i\phi_{0}},$$

with $\phi_{0}$ the mean direction and *ρ* the mean resultant length. The noise b(t) is assumed to be real Gaussian with 0 mean and variance $\sigma^{2}$.

### 3.2 S-transform

The analytic signal associated with our model is given by [43, Example 4.8]

(24) $$s_{n,a}(t)=\Omega_{n}e^{i(2\pi \nu_{0}t+\phi_{n})},\;n=1,\ldots ,N.$$

The S-transform of such a signal is given by (see Appendix C)

(25) $$T_{s_{n}}(t,f)=\Omega_{n}e^{-\frac{1}{2}{\left(2\pi \right)}^{2}{\left(1-\frac{\nu_{0}}{f}\right)}^{2}}e^{i\left[\phi_{n}-2\pi (f-\nu_{0})t\right]}.$$

The power is given by

(26) $${\left|T_{s_{n}}(t,f)\right|}^{2}=\Omega_{n}^{2}e^{-{\left(2\pi \right)}^{2}{\left(1-\frac{\nu_{0}}{f}\right)}^{2}}.$$

Its maximum is reached for $f=\nu_{0}$, with value equal to $\Omega_{n}^{2}$. Note that, had we defined the S-transform as the time-frequency transform of the real-valued signal instead of the analytic signal (as done, e.g., in [5]), $T_{s_{n}}(t,f)$ would have had a more complicated expression equal to about half the value found in (25), and the maximum of $|T_{s_{n}}(t,f){|}^{2}$ would be approximately equal to $\Omega_{n}^{2}/4$.

### 3.3 avgPOW

Incorporating (26) into (1) yields

(27) $${\mathrm{avgPOW}}_{s_{1:N}}(t,f)=e^{-{\left(2\pi \right)}^{2}{\left(1-\frac{\nu_{0}}{f}\right)}^{2}}\frac{1}{N}\sum_{n=1}^{N}\Omega_{n}^{2},$$

whose expectation is given by

(28) $$E\left[{\mathrm{avgPOW}}_{s_{1:N}}(t,f)\right]=\left(\Omega_{0}^{2}+\tau_{\Omega }^{2}\right)e^{-{\left(2\pi \right)}^{2}{\left(1-\frac{\nu_{0}}{f}\right)}^{2}}.$$

This quantity reaches its maximum for $f=\nu_{0}$, with value equal to $\Omega_{0}^{2}+\tau_{\Omega }^{2}$.

### 3.4 POWavg

From linearity of the time-frequency transform, $T_{\bar{s_{n}}}(t,f)$ is equal to the average of the $T_{s_{n}}(t,f)$'s, that is,

(29) $$T_{\bar{s_{n}}}(t,f)=e^{-\frac{1}{2}{\left(2\pi \right)}^{2}{\left(1-\frac{\nu_{0}}{f}\right)}^{2}}e^{-2i\pi (f-\nu_{0})t}\;\frac{1}{N}\sum_{n=1}^{N}\Omega_{n}\;e^{i\phi_{n}}.$$

Incorporating (29) into (2), we are led to

(30) $${\mathrm{POWavg}}_{s_{1:N}}(t,f)=e^{-{\left(2\pi \right)}^{2}{\left(1-\frac{\nu_{0}}{f}\right)}^{2}}{\left|\frac{1}{N}\sum_{n=1}^{N}\Omega_{n}e^{i\phi_{n}}\right|}^{2}$$

and corresponding expectation

(31) $$E\left[{\mathrm{POWavg}}_{s_{1:N}}(t,f)\right]=e^{-{\left(2\pi \right)}^{2}{\left(1-\frac{\nu_{0}}{f}\right)}^{2}}\;E\left[{\left|\frac{1}{N}\sum_{n=1}^{N}\Omega_{n}e^{i\phi_{n}}\right|}^{2}\right].$$

For large *N*, this quantity can be approximated by (see Appendix C)

(32) $$E\left[{\mathrm{POWavg}}_{s_{1:N}}(t,f)\right]=\Omega_{0}^{2}\rho^{2}e^{-{\left(2\pi \right)}^{2}{\left(1-\frac{\nu_{0}}{f}\right)}^{2}}+O\left(\frac{1}{N}\right),$$

where O(⋅) is the standard Bachmann–Landau notation. Note that we have the following relationship between avgPOW and POWavg:

(33) $$E\left[{\mathrm{POWavg}}_{s_{1:N}}(t,f)\right]\approx \frac{\Omega_{0}^{2}\rho^{2}}{\Omega_{0}^{2}+\tau_{\Omega }^{2}}E\left[{\mathrm{avgPOW}}_{s_{1:N}}(t,f)\right],$$

which is reminiscent of the relation found in [5], with the difference originating from the fact that we work with avgPOW instead of the average amplitude (avgAMP).

### 3.5 Numerical example

**Figure 1:**
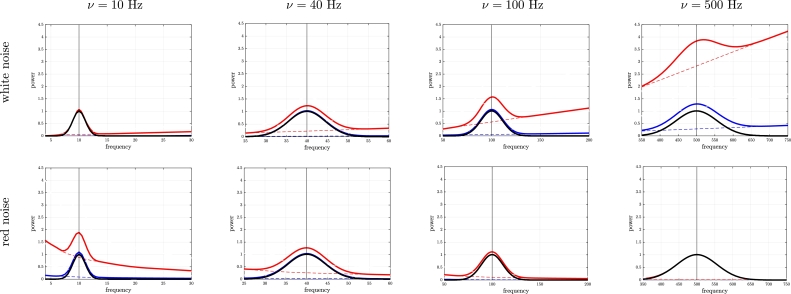
**Oscillatory signal.** Effect of noise on E(avgPOW) using the S-transform. We represented E[|*T*_*b*_(*t*,*f*)|^2^] (dashed lines) as well as E(avgPOW), either for the original signal *s*(*t*) (solid black line) or the noisy signal *x*(*t*) (solid colored lines) for color noise, either white (top) or red (bottom), and variance equal to either *σ*^2^ = 1 (blue) or *σ*^2^ = 10 (red). For red noise and *ν* = 500 Hz, the red and the black lines are superimposed.

We illustrate these results with the example of an oscillatory signal with frequency $\nu_{0}\in \{10,40,100,500\}$ Hz, amplitude $\Omega_{n}$ with mean $\Omega_{0}=1$ and standard deviation $\tau_{\Omega }=0.1$, mean resultant length ρ=0.25, sampling interval δt=0.5 ms, and N=300 trials. For noise, we considered two types of color Gaussian noise [35], [34], [61], [68]: white (corresponding to c=0) and red (corresponding to c=2). For each type of noise, we generated 1000 samples [68], computed the time-frequency transform of each sample, and approximated $E[|T_{b}(t,f){|}^{2}]$ by averaging the time-frequency transforms. We also used two levels of noise: moderate ($\sigma^{2}=1$) and high ($\sigma^{2}=10$). Results are summarized in Fig. 1 for avgPOW. Noise had no visible effect on the expectation of POWavg.

## 4 Simulation study

Time-frequency analysis of brain recordings from event-related protocols involving sensory stimulation has evidenced the presence of high-frequency oscillations (HFOs) ranging from 400 to 800 Hz in addition to the usual somatosensory evoked potential (SEP) [14], [15], [51], [63]. In the present section, we investigate the effect of noise on POWavg and avgPOW by generating synthetic data in this context.

### 4.1 Data generation

We generated signals on a time window of [−100,100] ms at a sampling rate of $f_{s}=2$ kHz (corresponding to a recording every δt=0.5 ms). Signals corresponding to the different trials were generated independently. For each trial *n*, we simulated an induced response in the [20,30] ms time window, and ongoing activity the rest of the time. Both the ongoing and the induced activities were generated using (22) with the same amplitude $\Omega_{n}$ and frequency $\nu_{n}$, but with different phase: $\phi_{n}^{\left(o\right)}$ for the ongoing activity, and $\phi_{n}^{\left(i\right)}$ for the induced activity. The exact values of parameters were sampled according to specific distributions (see Table 2). We added either white or red Gaussian noise with variance $\sigma^{2}\in \{1,2,5,10\}$ [68]. The signals were analyzed using the S-transform.

**Table 2:** **Simulation study.** Values of parameters for data generation.

Parameter	Distribution	Parameters	
Ω_*n*_	$N(\Omega_{0},\tau_{\Omega }^{2})$	Ω_0_ = 1	*τ*_Ω_ = 0.1
*ν*_*n*_	$N(\nu_{0},\tau_{\nu }^{2})$	*ν*_0_ = 500	*τ*_*ν*_ = 0
$\phi_{n}^{\left(o\right)}$	vonMises[*ϕ*_0_,*κ*^(*o*)^]	*ϕ*_0_ = 0	*κ*^(*o*)^ = 0
$\phi_{n}^{\left(i\right)}$	vonMises[*ϕ*_0_,*κ*^(*i*)^]	*ϕ*_0_ = 0	*κ*^(*i*)^ = 10

Since we are interested in oscillations in the high frequency range, we expected from previous calculations—see in particular (14), (15) and (21)—that the influence of noise would be (i) larger on avgPOW than on POWavg; and (ii) larger for white noise than for red noise.

### 4.2 Results

**Figure 2:**
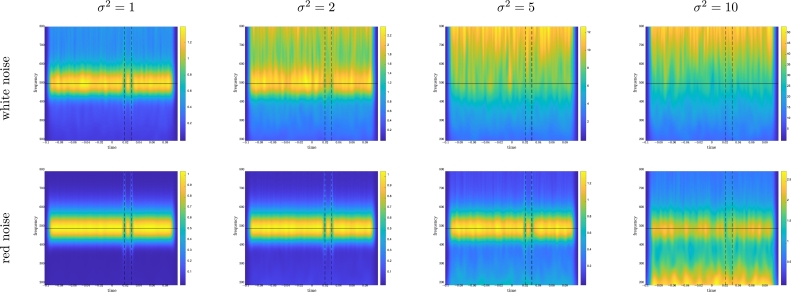
**Simulated data.** avgPOW for a 500 Hz oscillatory signal with white noise (top) or red noise (bottom), and *σ*^2^ ranging from 1 to 10. The color scales differ for all plots.

**Figure 3:**
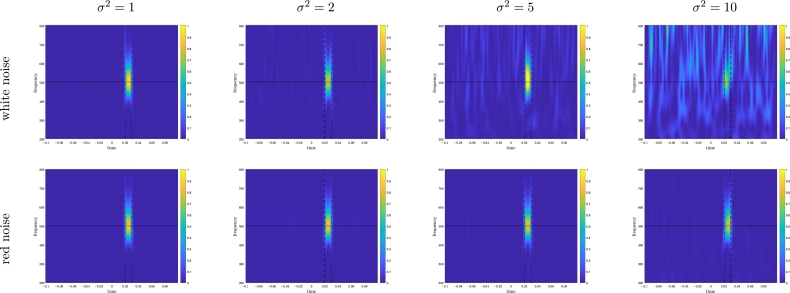
**Simulated data.** POWavg for a 500 Hz oscillatory signal with white noise (top) or red noise (bottom), and *σ*^2^ ranging from 1 to 10. The color scales are identical for all plots.

Results are illustrated on Fig. 2 for avgPOW and on Fig. 3 for POWavg. Without noise, avgPOW was able to visually enhance the presence of an oscillation around 500 Hz and POWavg the presence of a phase resetting. The effect of noise strongly depended on the type of noise and on the measure. Regarding the type of noise, white noise (corresponding to a linear increase in the time-frequency transform) was more visible than red noise (corresponding to a 1/f decrease in the time-frequency transform). This effect was all the more important that we were interested in a high-frequency phenomenon. Regarding the measure under consideration, we found that the effect of noise on POWavg was rather limited regardless of the type of noise and noise level. By contrast, avgPOW was more affected by noise than POWavg, and by white noise than by red noise.

## 5 Analysis of experimental data

We here investigate the effect of noise on EEG brain recordings acquired during an event-related protocol designed to generate somatosensory evoked potentials following median nerve stimulations in a healthy subject. To this end, we used the same dataset as in [5].

### 5.1 Data

Brain responses were acquired using multichannel EEG with a sampling frequency of 3 kHz. Electrical median nerve stimulation of 1 ms duration was applied to median nerve at the wrist level to elicit a burst of high-frequency oscillations (HFOs) in the 400–800 Hz frequency range superimposed onto the cortical N20 potential. The stimulus was applied 300 times, with a 500-ms inter-trial interval. Following previous recommendations [30], we studied the fronto-central channels (CP3–Fz). Data acquisition was performed at the Center for Neuroimaging Research (CENIR) of the Brain and Spine Institute (ICM, Paris, France). The experimental protocol was approved by the CNRS Ethics Committee (study #1402) and by the national ethical authorities (CPP Île-de-France, Paris 6 – Pitié-Salpêtrière and ANSM; ID-RCB 2015-A00462-47).

### 5.2 Analysis

The data was analyzed in two distinct time windows: [−200,−10] ms (before stimulus) and [10,200] ms (after stimulation). The peristimulus signal (in the window [−10,10] ms) was discarded to avoid stimulation-induced artifacts.

We first estimated both the prestimulus and poststimulus power spectral densities (PSDs) for each of the 300 trials using Welch's method [66]. Using the assumption that there was no structured and reproducible oscillations in the prestimulus window, we used the prestimulus data as a reference to assess the noise structure. For each prestimulus PSD, we performed a linear regression of its log over the frequency range 40–1000 Hz to estimate the type of color noise. The average of all estimated *c*'s over the 300 trials, denoted ${\overset{ˆ}{c}}_{\mathrm{PSDprestim}}$, was taken as a reference for all other cases (post-stimulus PSD as well as pre- and post-stimulus time-frequency transform).

As a second series of analyses, we applied time-frequency transform to both the prestimulus and poststimulus signals using the S-transform. We then computed avgPOW and POWavg. We also performed linear regression of the log of the measures as a function of the log of frequency in 40–1000 Hz, assuming a $1/f^{c-1}$ profile with $c={\overset{ˆ}{c}}_{\mathrm{PSDprestim}}$.

Finally, we focused on the HFOs in the [15,30] ms (poststimulus) time range and [600,1000] Hz frequency range. We compared various profiles with the expected $1/f^{c-1}$ profile with $c={\overset{ˆ}{c}}_{\mathrm{PSDprestim}}$.

### 5.3 Results

**Figure 4:**
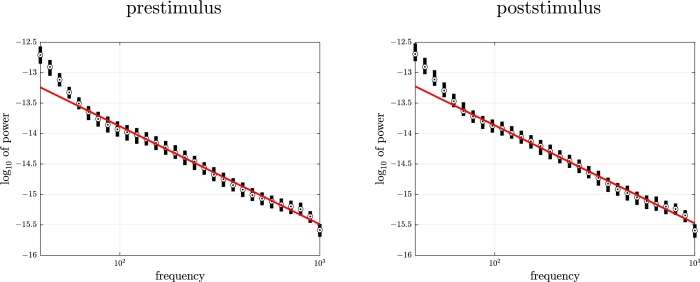
**Real data.** PSD analysis. Boxplot (median and [25%,75%] percentile interval) of prestimulus (left) and poststimulus (right) PSD corresponding to the signals of all trials together with expected profile of 1/*f*^*c*^ color noise with *c* = 1.61 (red line).

Results regarding the PSD are summarized in Fig. 4. Using the prestimulus data, we found that the signal exhibited a PSD that roughly decayed as $1/f^{c}$, with c≈1.610±0.117 (mean ± standard deviation over the 300 estimates), median of 1.61. Such range of values for *c* corresponds to color noise between pink noise (c=1) and red noise (c=2). The prestimulus PSD seemed to follow the $1/f^{c}$ trend with c=1.61 quite well, except for lower frequencies where larger than expected power density was found.

The value of ${\overset{ˆ}{c}}_{\mathrm{PSDprestim}}=1.61$ was used as a reference for both the poststimulus PSD as well as the pre- and poststimulus time-frequency transforms.

We observed that the poststimulus PSD behaved in fashion very similar to the prestimulus PSD. In particular, its profile also followed quite well a $1/f^{c}$ trend with $c={\overset{ˆ}{c}}_{\mathrm{PSDprestim}}$ for larger frequency values.

**Figure 5:**
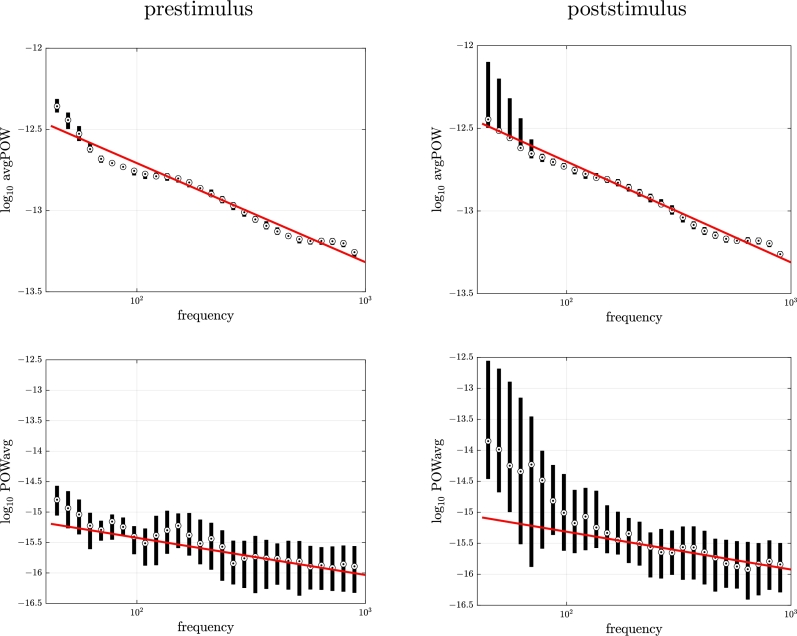
**Real data.** Time-frequency analysis of prestimulus (left) and poststimulus (right) signals. Boxplot (median and [25%,75%] interval) of avgPOW (top) and POWavg (bottom) together with expected profile 1/*f*^*c*−1^ profile (red line) with *c* = 1.61.

Results regarding the time-frequency transforms are illustrated in Fig. 5. While we observed no obvious structure in the prestimulus time-frequency transform with either avgPOW or POWavg, both poststimulus measures exhibited areas in the time-frequency domain with larger values before 50 ms, which are to be related to the somatosensory evoked response. This difference could also be seen in the boxplot of values across pre- and poststimulus windows, where both measures exhibited more variability in the lower frequency range poststimulus than prestimulus; also, values of POWavg were larger poststimulus than prestimulus. In all cases, we again observed a decent fit with the expected $1/f^{c-1}$ behavior, with $c={\overset{ˆ}{c}}_{\mathrm{PSDprestim}}$, in agreement with (21).

**Figure 6:**
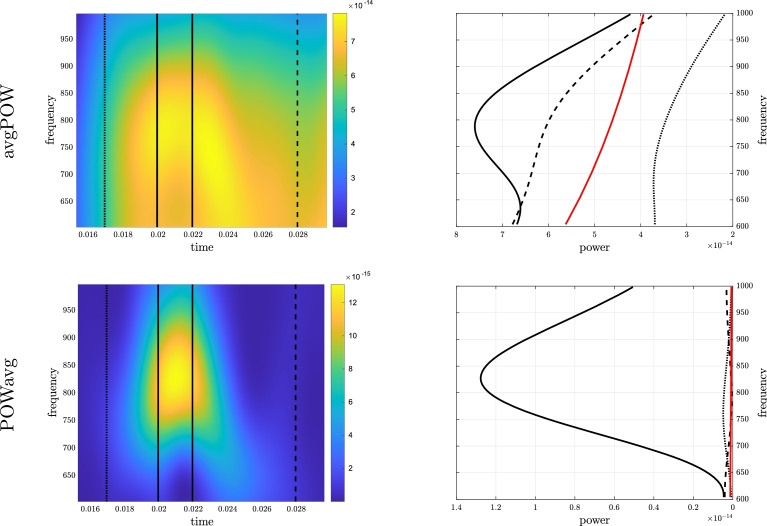
**Real data.** Analysis of HFO as observed by avgPOW (top) and POWavg (bottom). Left: Zoom of time-frequency transform for time in [15,30] ms and frequency in [600,1000] Hz window. Right: Mean frequency profile over [15,17] ms (dotted line), [20,22] ms (solid line), and [28,30] ms (dashed line) together with expected 1/*f*^*c*−1^ noise profile with *c* = 1.61 (red solid line).

A focus on the HFO is illustrated in Fig. 6. Compared to the expected profile of $1/f^{c-1}$ with $c={\overset{ˆ}{c}}_{\mathrm{PSDprestim}}$, both avgPOW and POWavg exhibited larger values in the 20–22 ms time range compared to the 15–17 ms and 28–30 ms time ranges. This effect was even more noticeable with POWavg than with avgPOW.

## 6 Discussion

In the present manuscript, we investigated the effect of noise on POWavg and avgPOW. More precisely, we assumed a model where additive Gaussian noise was added to the signal of interest and we compared the expected values of avgPOW and POWavg calculated either with or without noise. We showed that noise had an additive and positive effect on the expectations of the two measures. We also showed that the number of trials *N* had a different effect on POWavg and avgPOW: an influence on POWavg that decreased in 1/N, and an influence on avgPOW that did not depend on *N*. Since the influence of noise depends on its temporal structure (autocorrelation) and on the type of time-frequency transform used, we considered color noise (i.e., noise with a power spectral density proportional to $1/f^{c}$) analyzed with the S-transform. In that case, we showed that for both avgPOW and POWavg the expected effect of noise was on average proportional to $1/f^{c-1}$. We confirmed these general results in the case of a pure oscillatory signal with color noise, in the case of simulated data, as well as on experimental data.

The approach expounded in the present manuscript heavily relies on the time-frequency features of noise. As mentioned above, what is meant by the time-frequency transform of noise is not obvious. We circumvented this issue by taking a discrete perspective on noise, which allowed us to approximate integrals with Riemann sums. Since we deal with real-life signals, which are discrete by nature, this perspective does not lead to practical restrictions.

In this study, we also made different assumptions regarding the type of noise considered. In all cases, we focused on additive noise, as modeled in (8). The type of noise has a large impact on the global appearance of the time-frequency transform. We considered wide-sense stationary noise and investigated consequences of having color noise. In the case of color noise with a PSD of the form $1/f^{c}$, we showed that this 1/f structure could also be observed directly on the time-frequency diagrams. For instance, the expected profile for both avgPOW and POWavg in the case of the S-transform was in $1/f^{c-1}$. This key result entailed that the effect of noise on these two measures (i) increased with increasing frequencies for c<1; (ii) did not depend on frequency for c=1; and (iii) decreased with increasing frequencies for c>1. This effect was clearly visible on our calculations as well as on the simulation study and the analysis of experimental data. As a consequence, the effect of noise on our analyses critically depended on the frequency range of interest. For instance, in the case of HFOs, dealing with c<1 means that noise can potentially have a large impact on the measures. This effect globally remains the same in the case of time-frequency transform based on the continuous wavelet transform (see Section IV of Supplementary Material).

Signals with 1/f temporal autocorrelation structure have commonly been reported in EEG signal analysis [39], [40], [48], [6], [52], [55]. While the term “1/f noise” is quite common in the literature, the origin of the signal with 1/f power spectrum is more likely a signature of irregular and asynchronous neuronal activity, to be distinguished from rhythmic, oscillatory neural activity [47], [32], [31], [46], [19], [20], [26]. This non-oscillatory activity has been shown to be influenced by several factors, including age, sex, state, and task [9], [56], [16], [13], [50], [19], [10], [45], [49].

In the experimental data, we estimated the color (the value of *c* in $1/f^{c}$) from the prestimulus PSD. An alternative approach would be to use wavelet filtering [39]. This value of *c*, denoted ${\overset{ˆ}{c}}_{\mathrm{PSDprestim}}$, was found to be also a rather good indicator of the profile of the poststimulus PSD as well as the pre- and poststimulus values of avgPOW and POWavg—in particular for larger frequency values. This result gives weight to the underlying assumption of limited synchronized oscillatory activity in the experimental data.

In the present manuscript, we focused on the S-transform as a way to perform time-frequency analyses of brain signals for two reasons. First, it is a time-frequency transform that is commonly used in MEG/EEG data analysis. Furthermore, it has the advantage of rendering our calculations tractable. Another usual approach for time-frequency transform is the use of continuous wavelet transform [43]. A major difference between the S-transform and a continuous wavelet transform is that the S-transform uses a function whose $L_{1}$-norm is normalized (i.e., set to 1), while the continuous wavelet transform uses a function that is $L_{2}$-normalized. The consequences of this difference are twofold, depending on whether we focus on the signal of interest or the noise. From the perspective of the signal, the S-transform of a pure oscillatory signal of amplitude $\Omega_{0}$ and frequency $\nu_{0}$ yields a time-frequency transform whose maximum amplitude does not depend on $\nu_{0}$ and is equal to $\Omega_{0}$ at $f=\nu_{0}$ according to (25). By contrast, using the continuous wavelet transform would lead to a maximum amplitude that would be a decreasing function of amplitude (e.g., roughly in $1/\sqrt{f}$ for the Morlet wavelet). If we rather focus on the noise, the time-frequency transform of color noise was found to be of the order $1/f^{c-1}$, see (21). By contrast, it would be of order $1/f^{c}$ for a continuous wavelet transform. Altogether, both methods for time-frequency transform end up with the same signal-to-noise ratio. Still, what has been presented above as a prototypical behavior of the continuous wavelet transform might be undesirable, and some publications (such as [38]) and softwares (such as Matlab) use continuous wavelet transforms with $L_{1}$ normalization. In that case, the Morlet wavelet becomes very similar to the S-transform. See Section IV of Supplementary Material for more details on this issue.

We derived results regarding the expectations of avgPOW and POWavg. Using these results as indications regarding the behavior of the measures themselves amounts to neglecting their intrinsic variability. The validity of such an assumption depends on the measure. For POWavg, noise seemed to have a vanishing influence when the number of trials increases; neglecting variability might arguably make sense. By contrast, we observed that the influence of noise on avgPOW did not vanish, and ${\mathrm{avgPOW}}_{x_{1:N}}(t,f)$ did not become similar to ${\mathrm{avgPOW}}_{s_{1:N}}(t,f)$ as *N* increased. It might therefore be harder to do away with the residual variability in that case. A more precise quantification of the effect of noise on the variability of avgPOW and POWavg would involve the calculation of the variance of these measures.

In [5], we investigated the relationship between three measures from time-frequency transform, namely avgAMP, AMPavg, and inter-trial coherence (ITC) defined as [41]

(34) $$\mathrm{ITC}=\left|\frac{1}{N}\sum_{n=1}^{N}e^{i\arg\left[T_{x_{n}}(t,f)\right]}\right|.$$

Instead of working with avgAMP and AMPavg, we here rather considered avgPOW and POWavg, respectively. Obviously, both measures are related, but they are not equal: POWavg is equal to AMPavg^2^, while the relationship between avgPOW and avgAMP is more complex. The reason for the present choice of measures is that the effect of noise on avgPOW is easier to quantify on avgPOW and POWavg than it is on avgAMP and AMPavg.

Correction of 1/f noise has been deemed an important step in some analyses of brain recordings [19], [50], [20]. The fact that color noise with a 1/f structure translates into a time-frequency transform with a similar profile could be used to provide an efficient thresholding of the time-frequency diagram. Methods have been proposed for statistical hypothesis testing [57]. However, caution has to be exerted [7], [29], and the development of such a thresholding approach goes beyond the scope of the present manuscript.

Finally, we here considered the effect of noise on avgPOW and POWavg. We did not consider its effect on ITC. The reason for this choice is that noise has an effect on ITC that is qualitatively quite different from both avgPOW and POWavg. Determining the effect of noise on ITC is a research question in itself. While Cohen [12, §19.3] argues that noise should tend to increase ITC, van Diepen and Mazaheri [64] show evidence of a decrease of ITC with decreasing signal-to-noise. We would like to validate these statements and provide a derivation for ITC similar to what has been done for avgPOW and POWavg in the present manuscript. Only then can we start considering the effect of noise on the relationship obtained in [5].

## 7 Conclusion

In the present manuscript, we showed that additive noise tends on average to increase both avgPOW and POWavg. We quantified the main factors of influence for both measures, such as the noise variance, the number of trials, the sampling rate, the type of noise, and the frequency of interest. In particular:

• An increasing number of trials reduces the influence of noise on POWavg but not on avgPOW.
• The type of time-frequency transform (e.g., S-transform or continuous wavelet transform) has an influence on the way the time-frequency transforms for both the signal and the noise behave as a function of frequency.
• In the case of a color noise with PSD of the form $1/f^{c}$ analyzed with the S-transform, the relative effect of noise (i) increases with increasing frequency for c<1; (ii) does not depend on frequency for c=1; and (iii) decreases with increasing frequency for c>1.

These effects were established using theoretical calculations, simulation studies and analysis of experimental data. They can potentially have a large impact on the neuroscientist's work when dealing with time-frequency analysis. We hope the present results will help researchers improve their understanding and interpretation of time-frequency diagrams, as well as optimize their experimental setting based on their neuroscientific question. We specifically expect these results to hold relevance for non-invasive brain-computer interface (BCI) experiments using time-frequency analysis of event-related tasks. Maintaining an adequate signal-to-noise ratio is crucial in such contexts, as recordings may include not only brain signals such as EEG but also electromyogram (EMG) and other non-EEG artifacts. Our research should contribute to an improved quantification of the overall impact of these various forms of noise, which can significantly hinder BCI performance.

## CRediT authorship contribution statement

**Guillaume Marrelec:** Writing – original draft, Methodology, Formal analysis, Conceptualization. **Jonas Benhamou:** Writing – review & editing, Software, Methodology, Investigation, Data curation. **Michel Le Van Quyen:** Writing – review & editing, Methodology, Conceptualization.

## Declaration of Competing Interest

The authors declare that they have no known competing financial interests or personal relationships that could have appeared to influence the work reported in this paper.

## Appendix A Time-frequency transform of noise

We have

$$T_{x}^{\mathrm{RS}}(t,f)=\delta t\sum_{k}s(u_{k})\;\phi_{t,f}^{⁎}(u_{k})+\delta t\sum_{k}b(u_{k})\;\phi_{t,f}^{⁎}(u_{k})=T_{s}^{\mathrm{RS}}(t,f)+T_{b}^{\mathrm{RS}}(t,f).$$

This is true for any *δt*. Taking the limit δt→0, we obtain in particular

(35) $$T_{x}(t,f)=T_{s}(t,f)+T_{b}(t,f).$$

By taking the expectation of $T_{b}^{\mathrm{RS}}(t,f)$ from its definition, (10), and using the linearity of the expectation and the fact that the noise is of zero mean, we obtain

(36) $$E\left[T_{b}^{\mathrm{RS}}(t,f)\right]=\delta t\sum_{k}E\left[b(u_{k})\right]\;\phi_{t,f}^{⁎}(u_{k})=0.$$

Again, this is true for any *δt*; taking the limit δt→0, we obtain

(37) $$E\left[T_{b}(t,f)\right]=0.$$

## Appendix B Effect of noise on avgPOW and POWavg

From (1), we have

(38) $$E\left[{\mathrm{avgPOW}}_{x_{1:N}}(t,f)\right]=E\left[{\left|T_{x}(t,f)\right|}^{2}\right],$$

with $E\left[{\left|T_{x}(t,f)\right|}^{2}\right]$ given by

(39) $$E\left[{\left|T_{x}(t,f)\right|}^{2}\right]=E\left[{\left|T_{s}(t,f)\right|}^{2}\right]+E\left[{\left|T_{b}(t,f)\right|}^{2}\right]+2ℜ\left\{E\left[T_{s}(t,f)T_{b}(t,f)\right]\right\}.$$

Since s(t) and b(t) are independent, so are their time-frequency transforms. The expectation of their product is therefore equal to the product of their expectations,

(40) $$E\left[T_{s}(t,f)T_{b}(t,f)\right]=E\left[T_{s}(t,f)\right]E\left[T_{b}(t,f)\right],$$

which is equal to 0 from (13). We therefore obtain

(41) $$E\left[{\left|T_{x}(t,f)\right|}^{2}\right]=E\left[{\left|T_{s}(t,f)\right|}^{2}\right]+E\left[{\left|T_{b}(t,f)\right|}^{2}\right],$$

and

(42) $$E\left[{\mathrm{avgPOW}}_{x_{1:N}}(t,f)\right]=E\left[{\left|T_{s}(t,f)\right|}^{2}\right]+E\left[{\left|T_{b}(t,f)\right|}^{2}\right].$$

From (1), we see that the first expectation of the right-hand side of the equation is equal to ${\mathrm{avgPOW}}_{s_{1:N}}(t,f)$. In the end, $E\left[{\mathrm{avgPOW}}_{x_{1:N}}(t,f)\right]$ yields

(43) $$E\left[{\mathrm{avgPOW}}_{x_{1:N}}(t,f)\right]=E\left[{\mathrm{avgPOW}}_{s_{1:N}}(t,f)\right]+E\left[{\left|T_{b}(t,f)\right|}^{2}\right].$$

By an argument similar to the one used for avgPOW, we obtain that $E\left[{\mathrm{POWavg}}_{x_{1:N}}(t,f)\right]$ can be expressed as

(44) $$E\left[{\mathrm{POWavg}}_{x_{1:N}}(t,f)\right]=E\left[{\mathrm{POWavg}}_{s_{1:N}}(t,f)\right]+E\left[{\left|T_{\bar{b_{n}}}(t,f)\right|}^{2}\right].$$

We now need to explicitly calculate $E[|T_{\bar{b_{n}}}(t,f){|}^{2}]$. We use the linearity of the time-frequency transform and of the expectation, the fact that the $T_{b_{n}}(t,f)$'s are independent (because the $b_{n}$'s are), as well as $E[T_{b_{n}}(t,f)]=0$. We finally obtain

(45) $$E\left[{\left|T_{\bar{b_{n}}}(t,f)\right|}^{2}\right]=\frac{1}{N}E\left[{\left|T_{b}(t,f)\right|}^{2}\right].$$

Putting this result into Equation (44) finally yields for $E\left[{\mathrm{POWavg}}_{x_{1:N}}(t,f)\right]$

(46) $$E\left[{\mathrm{POWavg}}_{x_{1:N}}(t,f)\right]=E\left[{\mathrm{POWavg}}_{s_{1:N}}(t,f)\right]+\frac{1}{N}E\left[{\left|T_{b}(t,f)\right|}^{2}\right].$$

## Appendix C Oscillatory signal

The S-transform of the complex oscillatory signal $s(t)=\Omega e^{i(2\pi \nu t+\phi )}$ with amplitude Ω, frequency *ν* and phase *ϕ* is given by

(47) $$T_{s}(t,f)=\frac{\Omega |f|}{\sqrt{2\pi }}e^{i\phi }\int e^{-\frac{1}{2}f^{2}{\left(u-t\right)}^{2}}e^{2i\pi (\nu -f)u}\;du.$$

The integral is proportional to the value of the characteristic function of a Gaussian distribution with mean *t* and variance $1/f^{2}$ taken at value 2π(ν−f), which is equal to [33, Eq. (13.13)]

(48) $$e^{2i\pi (\nu -f)t-\frac{{\left[2\pi (\nu -f)\right]}^{2}}{2f^{2}}}.$$

The time-frequency transform is then given by

(49) $$T_{s}(t,f)=\Omega e^{-\frac{1}{2}{\left(2\pi \right)}^{2}{\left(1-\frac{\nu }{f}\right)}^{2}}e^{i\left[2\pi (\nu -f)t+\phi \right]}$$

and its power by

(50) $${\left|T_{s}(t,f)\right|}^{2}=\Omega^{2}e^{-{\left(2\pi \right)}^{2}{\left(1-\frac{\nu }{f}\right)}^{2}}.$$

Its maximum is reached for f=ν, with value equal to $\Omega^{2}$.

We have

(51) $$E\left[{\left|\frac{1}{N}\sum_{n=1}^{N}\Omega_{n}e^{i\phi_{n}}\right|}^{2}\right]=\frac{1}{N^{2}}\left[\sum_{n=1}^{N}E\left(\Omega_{n}^{2}\right)+\sum_{n\neq m}E\left(\Omega_{n}\Omega_{m}e^{i\phi_{n}}e^{-i\phi_{m}}\right)\right].$$

Independence of the $\Omega_{n}$'s and $\phi_{n}$'s leads to

(52) $$E\left(\Omega_{n}\Omega_{m}e^{i\phi_{n}}e^{-i\phi_{m}}\right)=E(\Omega_{n})E(\Omega_{m})E\left(e^{i\phi_{n}}\right)E\left(e^{-i\phi_{m}}\right)=E{\left(\Omega \right)}^{2}{\left|E\left(e^{i\phi }\right)\right|}^{2},$$

so that

(53) $$\sum_{n\neq m}E\left(\Omega_{n}\Omega_{m}e^{i\phi_{n}}e^{-i\phi_{m}}\right)=N(N-1)E{\left(\Omega \right)}^{2}{\left|E\left(e^{i\phi }\right)\right|}^{2}$$

and, from (51),

$$E\left[{\left|\frac{1}{N}\sum_{n=1}^{N}\Omega_{n}e^{i\phi_{n}}\right|}^{2}\right]=E{\left(\Omega \right)}^{2}{\left|E\left(e^{i\phi }\right)\right|}^{2}+\frac{1}{N}\left[E\left(\Omega^{2}\right)-E{\left(\Omega \right)}^{2}{\left|E\left(e^{i\phi }\right)\right|}^{2}\right]=E{\left(\Omega \right)}^{2}{\left|E\left(e^{i\phi }\right)\right|}^{2}+O\left(\frac{1}{N}\right).$$

## Appendix D Supplementary material

Supplementary Material The supplementary material includes various useful calculations and results regarding the S-transform, time-frequency transform of noise, as well as the Morlet continuous wavelet transform. Supplementary Material
