# Supplementary material for: Time-frequency analysis of event-related brain recordings: Effect of noise on power
Source: Heliyon. 2024 Sep 6;10(18):e35310. doi: 10.1016/j.heliyon.2024.e35310 (PMC11422058; doi:10.1016/j.heliyon.2024.e35310)
Supplement: Supplementary Material — The supplementary material includes various useful calculations and results regarding the S-transform, time-frequency transform of noise, as well as the Morlet continuous wavelet transform. [file mmc1.pdf]

Supplementary material for manuscript "Time-frequency analysis of event-related brain recordings: Effect of noise on power" by Marrelec et al.

Contents

1 Some basic results **2**

2 S-transform **2**

3 Time-frequency transform of noise **3**

3.1 Wide-sense stationary noise 3
3.2 Case of white noise 4
3.3 Case of color noise 5

4 Morlet wavelet **5**

4.1 Definition 5
4.2 Fourier transform 6
4.3 Relating location-scale and time-frequency 6
4.4 Transform of oscillatory signal 7
4.5 White noise 7
4.6 Color noise 8
4.7 avgPOW and POWavg for pure oscillatory signal 8
4.8 L_1-norm normalization 9

1 Some basic results

If K random variables $X_1, \ldots, X_K$ are independent, then the expectation of their product is equal to the product of their expectations (Anderson, 1958, §2.2.3)

$$\mathrm{E}\left(\prod_{k=1}^{K} X_k\right) = \prod_{k=1}^{K} \mathrm{E}\left(X_k\right). \tag{S-1}$$

A circular random variable is a random variable that, like an angle, is defined on the unit circle, i.e., whose value is only relevant modulo 2π. A key quantity for any circular random variable θ is its circular mean, defined as

$$\mathrm{E}\left(e^{i\theta}\right) = \int \mathrm{p}(\theta)\, e^{i\theta}\, \mathrm{d}\theta. \tag{S-2}$$

The argument of $\mathrm{E}\left(e^{i\theta}\right)$ is the mean angle or mean direction, while the modulus of $\mathrm{E}\left(e^{i\theta}\right)$ is the mean resultant length. It lays between 0 and 1 and is a measure of concentration of $\mathrm{p}(\theta)$.

A circular variable θ is said to follow a von Mises distribution with mean direction θ_0 and concentration parameter κ, denoted $\theta \sim \mathrm{VonMises}(\theta_0, \kappa)$, if its distribution is given by (Mardia and Jupp, 2000, §3.5.4)

$$\mathrm{p}(\theta) = \frac{1}{2\pi I_0(\kappa)} e^{\kappa \cos(\theta - \theta_0)},$$

where $I_0(\kappa)$ is the modified Bessel function of order 0,

$$I_0(\kappa) = \frac{1}{2\pi} \int_{-\pi}^{\pi} e^{\kappa \cos(\theta - \theta_0)}\, \mathrm{d}\theta. \tag{S-3}$$

The usual Gaussian distribution with mean μ and variance σ^2 is denoted by $\mathcal{N}(\mu, \sigma^2)$.

Finally, a function $f(N)$ is said to be $O(1/N)$ if it is bounded by a function proportional to $1/N$, that is, for which there exists an N_0 and a $k > 0$ such that

$$\forall N > N_0 \quad |f(N)| < \frac{k}{N}.$$

2 S-transform

The S-transform of a signal $s(t)$ is defined as (Stockwell et al., 1996)

$$T_s(t, f) = \frac{|f|}{\sqrt{2\pi}} \int s(u)\, e^{-\frac{1}{2} f^2 (u-t)^2} e^{-2i\pi f u}\, \mathrm{d}u. \tag{S-4}$$

We can express it as

$$T_s(t, f) = \int s(u) \phi_{t,f}(u)^*\, \mathrm{d}u \tag{S-5}$$

with

$$\phi_{t,f}(u) = \frac{|f|}{\sqrt{2\pi}}\, e^{-\frac{1}{2} f^2 (u-t)^2} e^{2i\pi f u}. \tag{S-6}$$

The Fourier transform of $\phi_{t,f}$ is given by

$$\widehat{\phi_{t,f}}(\nu) = \frac{|f|}{\sqrt{2\pi}} \int e^{-\frac{1}{2} f^2 (u-t)^2} e^{2i\pi (f-\nu) u}\, \mathrm{d}u. \tag{S-7}$$

This quantity can be understood as the value of the characteristic function of a normal distribution with mean t and variance $1/f^2$ calculated at point $2\pi(f - \nu)$, which is equal to (Johnson et al., 1994, Eq. (13.13))

$$\widehat{\phi_{t,f}}(\nu) = e^{-\frac{1}{2}(2\pi)^2 \left(1 - \frac{\nu}{f}\right)^2} e^{-2i\pi t(\nu - f)}. \tag{S-8}$$

The module of this quantity is given by $e^{-\frac{1}{2}(2\pi)^2\left(1-\frac{\nu}{f}\right)^2}$. For $f > 0$, we have

$$|\widehat{\phi_{t,f}}(\nu)| \leq e^{-\frac{1}{2}(2\pi)^2} \approx 2.73 \times 10^{-9}, \qquad \nu < 0.$$

Since this is very small, $\phi_{t,f}(u)$ is approximately an analytic function.

Consider now $f > 0$. Since $s(u)$ is real, it is easy to show that

$$T_s(t, -f) = T_s(t, f)^*. \tag{S-9}$$

Since $\phi_{t,f}(u)$ is approximately an analytic function and the signals we deal with are real, a common approach is to apply the S-transform to $s_a(u)$, the analytic signal associated to $s(u)$, defined as the signal whose Fourier transform is related to the original one by (Mallat, 1999, §4.3.2)

$$\widehat{s_a}(\nu) = \begin{cases} 2\widehat{s}(\nu) & \text{for } \nu \geq 0 \\ 0 & \text{for } \nu < 0, \end{cases} \tag{S-10}$$

where $\widehat{s}(\nu)$ is the Fourier transform of $s(u)$. Applying Plancherel theorem, we obtain for $T_{s_a}(t, f)$

$$\begin{aligned} \int s_a(u)\phi_{t,f}(u)^* \, \mathrm{d}u &= \int \widehat{s_a}(\nu)\widehat{\phi_{t,f}}(\nu)^* \, \mathrm{d}\nu \\ &= \underbrace{\int_{-\infty}^{0} \widehat{s_a}(\nu)\widehat{\phi_{t,f}}(\nu)^* \, \mathrm{d}\nu}_{=0} + \int_{0}^{+\infty} \underbrace{\widehat{s_a}(\nu)}_{=2\widehat{s}(\nu)} \widehat{\phi_{t,f}}(\nu)^* \, \mathrm{d}\nu \\ &= 2\int_{0}^{+\infty} \widehat{s}(\nu)\widehat{\phi_{t,f}}(\nu)^* \, \mathrm{d}\nu \\ &\approx 2\int \widehat{s}(\nu)\widehat{\phi_{t,f}}(\nu)^* \, \mathrm{d}\nu \\ &= 2\int s(u)\phi_{t,f}(u)^* \, \mathrm{d}u \\ &= 2T_s(t, f). \end{aligned}$$

As a conclusion, we have, for $f > 0$,

$$T_{s_a}(t, f) \approx 2T_s(t, f). \tag{S-11}$$

3 Time-frequency transform of noise

3.1 Wide-sense stationary noise

Since $\mathrm{E}[T_b(t, f)] = 0$, we have

$$\mathrm{Var}\left[T_b(t, f)\right] = \mathrm{E}\left[|T_b(t, f)|^2\right].$$

We express $|T_b(t, f)|^2$ as

$$\begin{aligned} |T_b(t, f)|^2 &= \left[\int b(u)\phi_{t,f}^*(u) \, \mathrm{d}u\right] \left[\int b(v)\phi_{t,f}^*(v) \, \mathrm{d}v\right]^* \\ &= \int b(u)b(v)\phi_{t,f}^*(u)\phi_{t,f}(v) \, \mathrm{d}u \, \mathrm{d}v. \end{aligned}$$

As a consequence, we obtain

$$\mathrm{E}\left[|T_b(t, f)|^2\right] = \int \mathrm{E}\left[b(u)b(v)\right] \phi_{t,f}^*(u)\phi_{t,f}(v) \, \mathrm{d}u \, \mathrm{d}v.$$

For a wide-sense stationary noise (i.e., a noise for which the mean and variance are time independent, and for which the autocorrelation function only depends on the lag between time points), Wiener–Khinchin theorem yields (Bendat and Piersol, 1986, §5.2)

$$\mathrm{E}\left[b(u)b(v)\right] = R_b(v-u) = \int S_b(\nu)e^{2i\pi\nu(v-u)}\mathrm{d}\nu, \tag{S-12}$$

where $S_b(\nu)$ is the power spectral density (PSD) of $b(t)$. We therefore have for $\mathrm{E}\left[|T_b(t,f)|^2\right]$

$$\begin{aligned}
\mathrm{E}\left[|T_b(t,f)|^2\right] &= \int\left[\iint S_b(\nu)e^{2i\pi\nu(v-u)}\mathrm{d}\nu\right]\phi^*_{t,f}(u)\phi^*_{t,f}(v)\,\mathrm{d}u\,\mathrm{d}v \\
&= \int S_b(\nu)\left[\iint\int \phi^*_{t,f}(u)\phi_{t,f}(v)e^{2i\pi\nu(v-u)}\,\mathrm{d}u\,\mathrm{d}v\right]\mathrm{d}\nu \\
&= \int S_b(\nu)\left[\iint \phi^*_{t,f}(u)e^{-2i\pi\nu u}\,\mathrm{d}u\right]\left[\iint \phi_{t,f}(v)e^{2i\pi\nu v}\,\mathrm{d}v\right]\mathrm{d}\nu \\
&= \int S_b(\nu)\,\widehat{\phi^*_{t,f}}(\nu)\,\widehat{\phi^*_{t,f}}(\nu)^*\mathrm{d}\nu \\
&= \int S_b(\nu)\left|\widehat{\phi^*_{t,f}}(\nu)\right|^2\mathrm{d}\nu \\
&= \int S_b(\nu)\left|\widehat{\phi_{t,f}}(\nu)\right|^2\mathrm{d}\nu.
\end{aligned} \tag{S-13}$$

3.2 Case of white noise

In the case of a Gaussian white noise, the power spectral density of $b(t)$ can be calculated through the autocorrelation function using the inverse relationship of Equation (S-12) (Bendat and Piersol, 1986, §5.2)

$$S_b(\nu) = \widehat{R_b}(\nu) = \int R_b(u)e^{-2i\pi\nu u}\,\mathrm{d}u. \tag{S-14}$$

The integral can be calculated using the approximation by the corresponding Riemann sum

$$S_b(\nu) \approx \delta t\sum_k R_b(k\delta t)e^{-2i\pi\nu k\delta t}, \tag{S-15}$$

with

$$R_b(0) = \mathrm{E}\left[b(t)^2\right] = \sigma^2, \tag{S-16}$$

and, for $k \neq 0$,

$$R_b(k\delta t) = \mathrm{E}\left[b(t)b(t+k\delta t)\right] = 0, \tag{S-17}$$

so that

$$S_b(\nu) = \sigma^2\delta t \tag{S-18}$$

and

$$\begin{aligned}
\mathrm{E}\left[|T_b(t,f)|^2\right] &= \sigma^2\,\delta t\int\left|\widehat{\phi_{t,f}}(\nu)\right|^2\mathrm{d}\nu \\
&= \sigma^2\,\delta t\int|\phi_{t,f}(u)|^2\,\mathrm{d}u.
\end{aligned} \tag{S-19}$$

The integral can be computed from the definition of $\phi_{t,f}(u)$ in the case of the S-transform, Equation (4) of the manuscript, as

$$\begin{aligned}
\int|\phi_{t,f}(u)|^2\,\mathrm{d}u &= \frac{2|f|^2}{\pi}\int e^{-f^2(u-t)^2}\mathrm{d}u \\
&= \frac{2\,|f|}{\sqrt{\pi}},
\end{aligned}$$

so that

$$\mathrm{E}\left[|T_b(t,f)|^2\right] = \frac{2|f|\sigma^2\delta t}{\sqrt{\pi}}. \tag{S-20}$$

3.3 Case of color noise

We assume that $b(t)$ has a PSD such that most of its energy is a domain where $S_b(\nu)$ is of the form

$$S_b(\nu) \propto \frac{1}{\nu^c}. \tag{S-21}$$

$\mathrm{E}[|T_b(t,f)|^2]$ can then be calculated from Equation (S-13), yielding

$$\mathrm{E}\left[|T_b(t,f)|^2\right] \quad \propto \quad \int \frac{1}{\nu^c}\left|\widehat{\phi_{t,f}}(\nu)\right|^2 \mathrm{d}\nu.$$

For the S-transform, we obtain

$$\mathrm{E}\left[|T_b(t,f)|^2\right] \propto \int \frac{1}{\nu^c}\, e^{-(2\pi)^2\left(1-\frac{\nu}{f}\right)^2}\, \mathrm{d}\nu. \tag{S-22}$$

Performing the parameter change $\nu \mapsto x = \nu/f$, we are led to

$$\begin{aligned}\mathrm{E}\left[|T_b(t,f)|^2\right] \quad &\propto \quad \frac{1}{f^{c-1}}\int \frac{1}{x^c} e^{-(2\pi)^2(1-x)^2}\mathrm{d}x \\ &\propto \quad f^{-(c-1)}.\end{aligned}$$

4 Morlet wavelet

4.1 Definition

The continuous wavelet transform with Morlet wavelet for a scale a and shift b is defined as

$$\int s(u)\, \psi^*_{a,b}(u)\, \mathrm{d}u \tag{S-23}$$

with

$$\psi_{a,b}(u) = \frac{1}{\sqrt{a}}\psi\left(\frac{u-b}{a}\right) \tag{S-24}$$

and

$$\psi(u) = c_r \pi^{-\frac{1}{4}} e^{-\frac{u^2}{2}}\left(e^{iru} - d_r\right), \tag{S-25}$$

where r is a parameter, and d_r and c_r are defined by

$$d_r = e^{-\frac{1}{2}r^2} \quad \text{and} \quad c_r = \left(1 + e^{-r^2} - 2e^{-\frac{3}{4}r^2}\right)^{-\frac{1}{2}}. \tag{S-26}$$

ψ has the following properties: its L_2-norm is equal to 1 and its integral is equal to 0. For r large enough, d_r is small (e.g., for $r > 5$, $d_r < 4 \times 10^{-6}$) and can be neglected, leading to the approximate form of the Morlet transform

$$\psi(u) = \pi^{-\frac{1}{4}} e^{-\frac{1}{2}u^2} e^{iru}. \tag{S-27}$$

For reasons that will become clear below, we set $r = 2\pi q$, so that $\psi(u)$ can be expressed as

$$\psi(u) = \pi^{-\frac{1}{4}} e^{-\frac{1}{2}u^2} e^{2i\pi q u}, \tag{S-28}$$

and $\psi_{a,b}(u)$ as

$$\psi_{a,b}(u) = \frac{\pi^{-\frac{1}{4}}}{\sqrt{a}} e^{-\frac{(u-b)^2}{2a^2}} e^{2i\pi q\frac{u-b}{a}}. \tag{S-29}$$

4.2 Fourier transform

The Fourier transform of $\psi_{a,b}(u)$ is given by

$$\begin{aligned}\widehat{\psi_{a,b}}(\nu) &= \int \psi_{a,b}(u)\, e^{-2i\pi\nu u}\,\mathrm{d}u \\ &= \frac{\pi^{-\frac{1}{4}}}{\sqrt{a}} \int e^{-\frac{(u-b)^2}{2a^2}} e^{2i\pi\left(q\frac{u-b}{a}-\nu u\right)}\,\mathrm{d}u \\ &= \pi^{\frac{1}{4}}\sqrt{2a}e^{-2i\pi\frac{q}{a}b}\left[\frac{1}{\sqrt{2\pi a^2}}\int e^{-\frac{(u-b)^2}{2a^2}} e^{2i\pi\left(\frac{q}{a}-\nu\right)u}\,\mathrm{d}u\right].\end{aligned}$$

The term between brackets is the characteristic function of a Gaussian distribution with mean b and variance a^2 taken at value $2\pi(\frac{q}{a}-\nu)$, which is equal to (Johnson et al., 1994, Eq. (13.13))

$$e^{2i\pi\left(\frac{q}{a}-\nu\right)b-\frac{1}{2}(2\pi)^2a^2\left(\frac{q}{a}-\nu\right)^2}.$$

In the end, we obtain

$$\widehat{\psi_{a,b}}(\nu) = \pi^{\frac{1}{4}}\sqrt{2a}e^{-\frac{1}{2}(2\pi)^2a^2\left(\frac{q}{a}-\nu\right)^2}e^{-2i\pi\nu}. \tag{S-30}$$

Its power is given by

$$|\widehat{\psi_{a,b}}(\nu)|^2 = 2a\sqrt{\pi}e^{-(2\pi)^2a^2\left(\frac{q}{a}-\nu\right)^2}. \tag{S-31}$$

The central frequency $\nu_{\max}$ of $\psi_{a,b}(u)$, which is the frequency for which $|\widehat{\psi_{a,b}}(\nu)|^2$ is maximal, is given by $\nu_{\max} = q/a$. This is independent of b. In particular, q is the central frequency of $\psi(u)$.

4.3 Relating location-scale and time-frequency

Equation (S-23) is of the form of Equation (3) of the manuscript with $\phi_{t,f}(u) = \psi_{a,b}(u)$. To specify this relationship, we need to relate (t, f) and (a, b). If we set $b = t$ and $a = q/f$ (i.e., $f = q/a$), we obtain

$$\phi_{t,f}(u) = \psi_{\frac{q}{f},t}(u) = \pi^{-\frac{1}{4}}\sqrt{\frac{f}{q}}e^{-\frac{1}{2}\left(\frac{f}{q}\right)^2(u-t)^2}e^{2i\pi f(u-t)}, \tag{S-32}$$

with power

$$|\phi_{t,f}(u)|^2 = \frac{f}{q\sqrt{\pi}}e^{-\left(\frac{f}{q}\right)^2(u-t)^2}. \tag{S-33}$$

We see that the maximum of $|\phi_{t,f}(u)|^2$ is reached for $u = t$. From Equation (S-30), the Fourier transform is given by

$$\widehat{\phi_{t,f}}(\nu) = \pi^{\frac{1}{4}}\sqrt{\frac{2q}{f}}e^{-\frac{1}{2}(2\pi)^2q^2\left(1-\frac{\nu}{f}\right)^2}e^{-2i\pi\nu t}, \tag{S-34}$$

with power equal to

$$|\widehat{\phi_{t,f}}(\nu)|^2 = \frac{2q\sqrt{\pi}}{f}e^{-(2\pi)^2q^2\left(1-\frac{\nu}{f}\right)^2}. \tag{S-35}$$

The maximum of this quantity is reached for $\nu = f$. The time-frequency transform corresponding to the fonction $\phi_{t,f}(u)$ defined Equation (S-32) then reads

$$T_s(t,f) = \pi^{-\frac{1}{4}}\sqrt{\frac{f}{q}}e^{2i\pi ft}\int s(u)\,e^{-\frac{1}{2}\left(\frac{f}{q}\right)^2(u-t)^2}e^{-2i\pi fu}\,\mathrm{d}u. \tag{S-36}$$

4.4 Transform of oscillatory signal

The (approximate) Morlet transform of a complex oscillatory signal of the form $s(u) = \Omega_0 e^{i(2\pi\nu_0 u+\phi_0)}$, i.e., with amplitude Ω_0, frequency ν_0 and phase ϕ_0, is given by

$$\begin{aligned}
&\Omega_0 \pi^{-\frac{1}{4}} \sqrt{\frac{f}{q}} e^{i(2\pi f t+\phi_0)} \int e^{-\frac{1}{2}\left(\frac{f}{q}\right)^2 (u-t)^2} e^{2i\pi(\nu_0-f)u} \,\mathrm{d}u \\
= \;& \Omega_0 \pi^{\frac{1}{4}} \sqrt{\frac{2q}{f}} e^{i(2\pi f t+\phi_0)} \left[\frac{1}{\sqrt{2\pi\left(\frac{q}{f}\right)^2}} \int e^{-\frac{1}{2}\left(\frac{f}{q}\right)^2 (u-t)^2} e^{2i\pi(\nu_0-f)u} \,\mathrm{d}u \right]. \qquad \text{(S-37)}
\end{aligned}$$

The term in brackets is the characteristic function of a Gaussian distribution with mean t and variance q^2/f^2 calculated at value $2\pi(\nu_0 - f)$, which is equal to (Johnson et al., 1994, Eq. (13.13))

$$e^{2i\pi(\nu_0-f)t} e^{-\frac{1}{2}(2\pi)^2\left(\frac{q}{f}\right)^2(\nu_0-f)^2}.$$

In the end, we obtain

$$T_s(t,f) = \Omega_0 \pi^{\frac{1}{4}} \sqrt{\frac{2q}{f}} e^{-\frac{1}{2}(2\pi)^2 q^2 \left(1-\frac{\nu_0}{f}\right)^2} e^{i(2\pi\nu_0 t+\phi)}. \qquad \text{(S-38)}$$

The power of this quantity is given by

$$|T_s(t,f)|^2 = \Omega_0^2 \frac{2q\sqrt{\pi}}{f} e^{-(2\pi)^2 q^2 \left(1-\frac{\nu_0}{f}\right)^2}, \qquad \text{(S-39)}$$

which is maximal for

$$f = 2\pi^2 q^2 \nu_0 \left(\sqrt{1+\frac{1}{\pi^2 q^2}} - 1\right) = \alpha\nu_0, \qquad \text{(S-40)}$$

where we set

$$\alpha = 2\pi^2 q^2 \left(\sqrt{1+\frac{1}{\pi^2 q^2}} - 1\right). \qquad \text{(S-41)}$$

For $\pi^2 q^2 \gg 1$, we have $\alpha \approx 1$, i.e., the maximum is reached for f close to ν_0. The value at $f = \alpha\nu_0$ is given by

$$|T_s(t,\alpha\nu_0)|^2 = \Omega_0^2 \frac{2q\sqrt{\pi}}{\alpha\nu_0} e^{-(2\pi)^2 q^2 \left(1-\frac{1}{\alpha}\right)^2}. \qquad \text{(S-42)}$$

This quantity decreases in $1/\nu_0$.

4.5 White noise

In the case of white noise with variance σ^2, we have according to Equation (18) of the manuscript

$$\mathrm{E}\left[|T_b(t,f)|^2\right] = \sigma^2\,\delta t \int |\phi_{t,f}(u)|^2 \,\mathrm{d}u = \sigma^2\,\delta t, \qquad \text{(S-43)}$$

since the Morlet wavelet is L_2-normalized to 1. This expectation does not depend on f—compare with Equation (19) of the manuscript.

4.6 Color noise

According to Equation (20) of the manuscript and Equation (S-35), we have

$$\mathrm{E}\left[|T_b(t,f)|^2\right] \propto \frac{1}{f}\int \frac{1}{\nu^c}\, e^{-(2\pi)^2 q^2 \left(1-\frac{\nu}{f}\right)^2}\, \mathrm{d}\nu. \tag{S-44}$$

Performing the parameter change $\nu \mapsto x = \nu/f$, we are led to

$$\mathrm{E}\left[|T_b(t,f)|^2\right] \propto f^{-c}. \tag{S-45}$$

In this case, the variance of the noise time-frequency transform decays as fast as its power spectral density. Again, the result obtained for white noise, Equation (S-43), is compatible with this result with $c = 0$ (which corresponds to white noise).

4.7 avgPOW and POWavg for pure oscillatory signal

We consider purely oscillatory signals as in §3 of the manuscript. From Equation (1) of the manuscript and Equation (S-39) and , we obtain

$$\mathrm{avgPOW}_{s_{1:N}}(t,f) = \frac{2q\sqrt{\pi}}{f} e^{-(2\pi)^2 q^2 \left(1-\frac{\nu_0}{f}\right)^2} \frac{1}{N}\sum_{n=1}^{N} \Omega_n^2, \tag{S-46}$$

with expectation given by

$$\mathrm{E}\left[\mathrm{avgPOW}_{s_{1:N}}(t,f)\right] = \frac{2q\sqrt{\pi}}{f}\left(\Omega_0^2 + \tau_\Omega^2\right) e^{-(2\pi)^2 q^2 \left(1-\frac{\nu_0}{f}\right)^2}. \tag{S-47}$$

Also, from Equations (2) and (7) of the manuscript as well as Equation (S-38), we are led to

$$\mathrm{POWavg}_{s_{1:N}}(t,f) = \frac{2q\sqrt{\pi}}{f} e^{-(2\pi)^2 q^2 \left(1-\frac{\nu_0}{f}\right)^2} \left|\frac{1}{N}\sum_{n=1}^{N} \Omega_n e^{i\phi_n}\right|^2, \tag{S-48}$$

and corresponding expectation

$$\mathrm{E}\left[\mathrm{POWavg}_{s_{1:N}}(t,f)\right] = \frac{2q\sqrt{\pi}}{f} e^{-(2\pi)^2 q^2 \left(1-\frac{\nu_0}{f}\right)^2}\, \mathrm{E}\left[\left|\frac{1}{N}\sum_{n=1}^{N} \Omega_n e^{i\phi_n}\right|^2\right]. \tag{S-49}$$

We then use the fact that the expectation of the term within brackets can be approximated by (see Appendix C of the manuscript)

$$\mathrm{E}\left[\left|\frac{1}{N}\sum_{n=1}^{N} \Omega_n e^{i\phi_n}\right|^2\right] = \Omega_0^2 \rho^2 + O\left(\frac{1}{N}\right). \tag{S-50}$$

Finally,

$$\mathrm{E}\left[\mathrm{POWavg}_{s_{1:N}}(t,f)\right] = \frac{2q\sqrt{\pi}}{f} \Omega_0^2 \rho^2 e^{-(2\pi)^2 q^2 \left(1-\frac{\nu_0}{f}\right)^2} + O\left(\frac{1}{N}\right). \tag{S-51}$$

4.8 L_1-norm normalization

We here compute the normalization constant for a Morlet wavelet with L_1 normalization, $\phi_{t,f}^{(L_1)}(u)$. From Equation (S-32), we have

$$\phi_{t,f}^{(L_1)}(u) \propto e^{-\frac{1}{2}\left(\frac{f}{q}\right)^2 (u-t)^2} e^{2i\pi f(u-t)}, \tag{S-52}$$

In the case of an L_1-norm normalization, we require

$$\int \left|\phi_{t,f}^{(L_1)}(u)\right| \mathrm{d}u = 1. \tag{S-53}$$

The absolute value of the right-hand side of Equation (S-52),

$$e^{-\frac{1}{2}\left(\frac{f}{q}\right)^2 (u-t)^2}, \tag{S-54}$$

is positive and proportional to a Gaussian distribution with mean t and variance $(q/f)^2$. As a consequence,

$$\int e^{-\frac{1}{2}\left(\frac{f}{q}\right)^2 (u-t)^2} \mathrm{d}u = \sqrt{2\pi\left(\frac{q}{f}\right)^2} = \frac{q\sqrt{2\pi}}{|f|}. \tag{S-55}$$

From there, we obtain

$$\phi_{t,f}^{(L_1)}(u) = \frac{|f|}{q\sqrt{2\pi}} e^{-\frac{1}{2}\left(\frac{f}{q}\right)^2 (u-t)^2} e^{2i\pi f(u-t)}. \tag{S-56}$$

Comparing this result with Equation (S-6), we see that the difference with the S-transform is twofold:

- In the amplitude of $\phi_{t,f}(u)$, the frequency f (in the case of the S-transform) is changed to f/q (in the case of the Morlet wavelet);
- The phase, which is equal to $2\pi fu$ for the S-transform, is shifted so that it is equal to 0 for $u = t$.

References

T. W. Anderson. *An Introduction to Multivariate Statistical Analysis.* Wiley Publications in Statistics. John Wiley and Sons, New York, 1958.

J. S. Bendat and A. G. Piersol. *Random Data. Analysis and Measurement Procedures.* John Wiley & Sons, New York, 2nd edition, 1986.

N. L. Johnson, S. Kotz, and N. Balakrishnan. *Continuous Univariate Distributions*, volume 1 of *Wiley Series in Probability and Mathematical Statistics: Applied Probability and Statistics Section.* John Wiley and Sons, New York, 2nd edition, 1994.

S. Mallat. *A Wavelet Tour of Signal Processing.* Wavelet Analysis & Its Applications. Academic Press, Amsterdam, 2nd edition, 1999.

K. V. Mardia and P. E. Jupp. *Directional Statistics.* Wiley Series in Probability and Statistics. Wiley, Chichester, 2000.

R. G. Stockwell, L. Mansinha, and R. P. Lowe. Localization of the complex spectrum: the S transform. *IEEE Transactions on Signal Processing*, 44(4):998–1001, 1996.
